# 46 XX Ovotesticular Disorder of Sex Development with Gonadotropin-Releasing Hormone Receptor, Autosomal Recessive Heterozygous Missense Mutation and Autosomal Dominant Heterozygous Missense Mutation of the *PROKR2* Gene: A Case Report

**DOI:** 10.1055/s-0044-1788060

**Published:** 2024-07-09

**Authors:** Francesca Peranzoni, Roberto De Castro, Emilio Merlini, Yen Le Nguyen

**Affiliations:** 1 Department of Pediatric Surgery, Lausanne University Hospital, Lausanne, Switzerland; 2 Thien Nhan and Friends Association, Italy; 3 Department of Pediatric Surgery, Hospital of Alexandria, Italy; 4 Department of Pediatric Urology, Vietnam National Hospital of Pediatric 2, Ho Chi Minh City, Vietnam

**Keywords:** ovotestis, Kallmann syndrome, disorder of sex development

## Abstract

True hermaphroditism is a disorder of sex development (DSD), accounting for less than 5% of all DSD cases, defined by the simultaneous presence of testicular tissue and ovarian tissue in the same individual. In the reported case, the patient presented two genetic mutations involved in the pathogenic pathway of the DSD condition associated with the clinical features of Kallmann syndrome (KS), a developmental disease that associates hypogonadotropic hypogonadism (HH), due to gonadotropin-releasing hormone deficiency, and anosmia, related to the absence or hypoplasia of the olfactory bulbs. Given the variable degree of hyposmia in KS, the distinction between KS and normosmic idiopathic HH is currently unclear, especially as HH patients do not always undergo detailed olfactory testing. This syndrome is very rare, with an estimated prevalence of 1:80,000 in males and 1:40,000 in females.

This is the only case report concerning a patient with 46 XX true hermaphroditism affected by HH and digenic inheritance of Kallmann syndrome.

## Introduction

True hermaphroditism is a disorder of sex development (DSD), accounting for less than 5% of all DSD cases, defined by the simultaneous presence of testicular tissue and ovarian tissue in the same individual. Kallmann syndrome (KS) is characterized by a developmental disease that associates hypogonadotropic hypogonadism (HH), with gonadotropin-releasing hormone (GnRH) deficiency, and anosmia, relative to the absence or hypoplasia of the olfactory bulbs. This syndrome has an estimated prevalence of 1:80,000 in males and 1:40,000 in females. 1

Here we report the case of a patient with a 46 XX karyotype, gonadotropin-releasing hormone receptor (GnRHR), and PROKR2 mutation, diagnosed with true hermaphroditism who underwent genital reconstructive surgery during a humanitarian mission in Vietnam in autumn 2023.

## Case Report

The patient, the first child of a nonconsanguineous couple, was referred to the Vietnam National Hospital of Pediatric 2 in Ho Chi Minh City immediately after birth due to ambiguous external genitalia. Family history and pregnancy anamnesis showed no particularities.

**Fig. 1:**
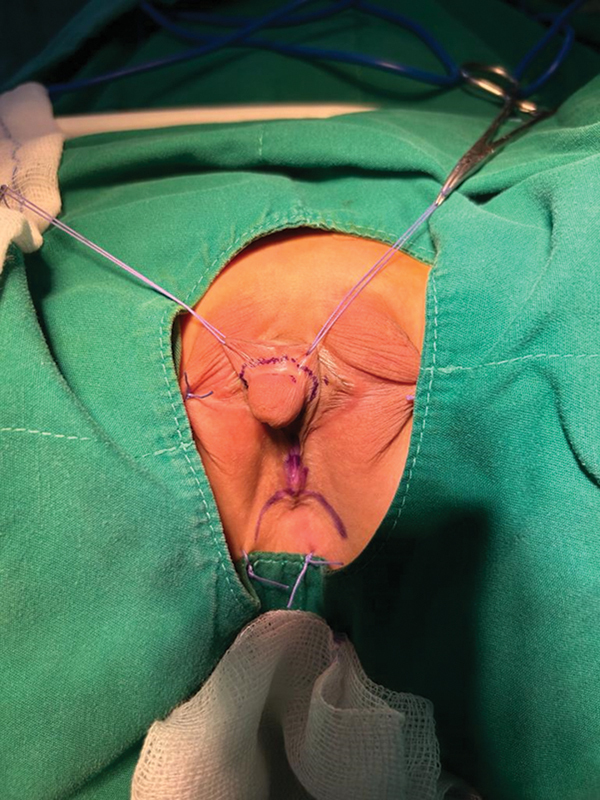
Phenotypical appearance.

At the objective examination of the external genitalia, an ovalar structure was palpable in the left labioscrotal area. The collateral side was empty. Only one orifice, compatible with a proximal hypospadias or a urogenital sinus, was present in the perineal area. Anus was in the correct position as well as the anal sphincter ( Fig. 1 ).

An ultrasound was realized and confirmed the presence of an ovalar, vascularized structure in the left labioscrotal region, an abdominal ultrasound excluded the presence of intraabdominal Mullerian structure as well as the normality of other abdominal organs. A blood test sample showed normal electrolytes Na (138 mmol/L), K 4.75 mmol/L, Ca 2.49 mmol/L, Cl 107.5 mmol/L, and morning cortisol level between normal margins 30.7 pg/mL. The aldosterone was normal at 20.66 ng/dL, active renin was slightly higher at 43.12 ng/L, and beta-human choriogonadotropin (hCG) was normal at <1.20 mUL/mL. Testosterone was 47.26 ng/dL, which is a normal level for a female but a lower level for a male. Dehydroepiandrosterone (DHEASO4) was also normal.

A karyotype examination was realized and an endocrinological investigation was done. The child has confirmed 46 XX through a genetic karyotype analysis with the fluorescence in situ hybridization technique using the kit Cytocell SRY/DYZ1/DXZ1 to identify the SRY segment. The chromosome analysis was done through a Kit NEBNext (United States) and then the new generation sequencing was realized with the system NextSeq, Illumina (United States), the control reference DNA (hg19) was split in the region of 100 kb to detect the copy variations numbers defined according to the following database: http://decipher.sanger.ac.uk/ , http://dgv.tcag.ca./ , http://www.internationalgenome.org/ , http://omim.org/ . In conclusion, no CVN was detected. As a routine examination of the sequencing of the congenital adrenal hyperplasia, the sequencing of the CYP21A2 was realized and was normal.

Exploration laparoscopy was performed when the child was 2 years old; meanwhile, two biopsies of the ovalar structure were performed and the axis of the structure in the left labioscrotal region was realized. The absence of intra-abdominal Mullerian remnant was confirmed. A few months later endoscopic examination of the urogenital sinus was realized and showed one vagina and one bladder with a common channel 3 cm long.

Biopsies, fixed in hematoxylin–eosin, revealed the presence of ovarian tissue. A new karyotype was realized. The result still confirmed a 46 XX. Genetic studies further confirmed the absence of the *SRY* gene, and the absence of mutation of the CYP21A2 gene and identified the mutation of two genes: GnRHR, autosomal recessive, heterozygous mutation missense, encoding for hypogonadism hypogonadal 7 without anosmia, and PROKR2 autosomal dominant missense mutation responsible for hypogonadism hypogonadotropic 3 with or without anosmia.

According to these considerations and the female acting-like attitude, the patient underwent a female genital reconstruction with reduction clitoridoplasty and labioplasty at 6 years of age.

**Fig. 2:**
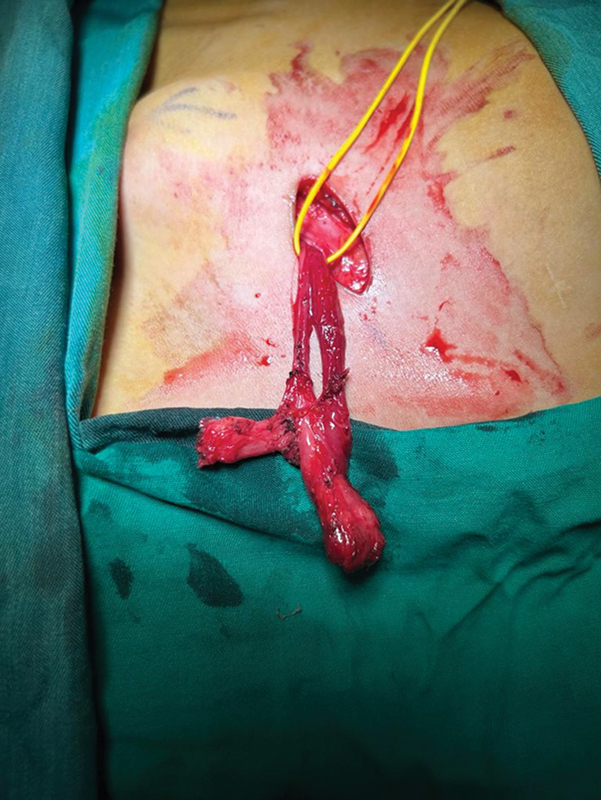
Ovotestis.

**Fig. 3:**
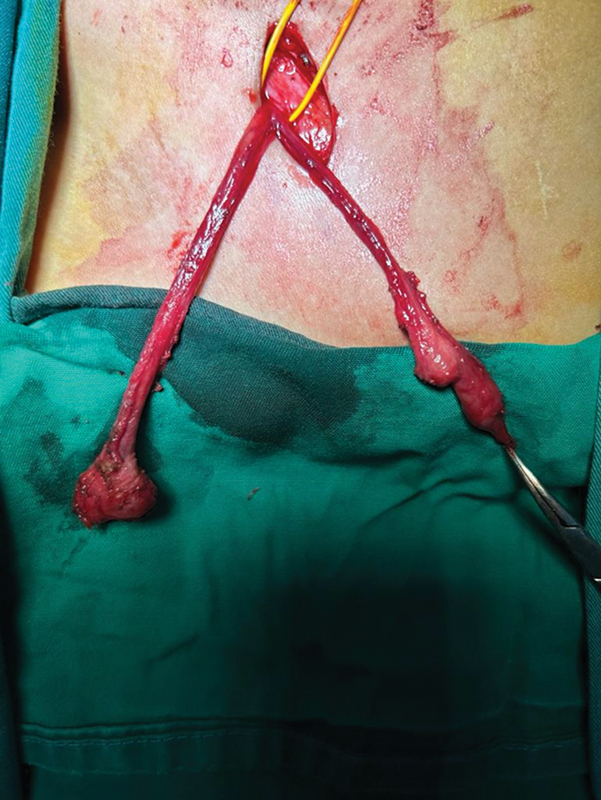
Splitted ovotestis, on the right the testis and the ovary in the left.

The previous fixed structure in the left labia was reached through an inguinal approach, at that moment a structure compatible with ovotestis was found ( Fig. 2 ). The structure was further split ( Fig. 3 ) and the testis was removed and sent to anatomy pathology for histological examination. The remnant ovary was pulled into the abdominal cavity through the inguinal ring. The specimen, fixed with hematoxylin and eosin, showed a testis structure with spermatic ducts and vas deferens melted with fibrotic tissue.

The patient was discharged 2 weeks after the surgery.

At 6-month follow-up, the patient had no complaints, and the neovagina presented a good healing process.

## Discussion

In human fetuses during the ambisexual stage, primordial germ cells migrate from their site of origin in the allantois into the genital ridges around week 5. Sex cords are formed within the gonads in the sixth embryonic week, and the urogenital folds and labioscrotal folds begin to form in the perineum. Neurons that produce GnRH develop from the epithelium of the medial olfactory pit and move to the fetal hypothalamus by migrating along nerve fibers. 2 This process occurs at approximately 40 days of gestation. 3 The pituitary gland develops and begins synthesizing both luteinizing hormone (LH) and follicle-stimulating hormone (FSH) at 9 weeks of gestation. 4

The Mullerian and Wolffian ducts form around the 7th embryonic week and gonadal differentiation occurs from the 10th through 12th embryonic weeks.

The Sex-determining Region of the Y chromosome ( *SRY* ) gene is required to initiate signaling for male gonadal differentiation. 5 *SRY* gene expression results in the differentiation of Sertoli cells, responsible for Mullerian-inhibiting substance production. The fetal testicles also express FSH receptors that probably control Sertoli cell proliferation, although only a few studies exist regarding the effects of FSH. 6 7 SRY also causes the differentiation of Leydig cells.

During the first trimester of gestation, placental hCG induces the differentiation of Leydig cells and stimulates testosterone (T) production through the activation of the LH/CG receptors expressed on their surfaces. After this period T secretion comes under the control of pituitary LH. 8 T permits the persistence of the Wolffian duct and its differentiation into the epididymis, vas deferens, and seminal vesicles.

5α-reductase is an enzyme that converts testosterone to dihydrotestosterone. This androgen drives the growth of the external genitalia and prostate. 9

Additional genes involved in the gonadal genesis and normal SRY levels of expression are GATA4, FOG2, 10 and SRY box-related gene 9. 11

The absence of the *SRY* gene results in feminine differentiation. In females, the Mullerian ducts persist and develop into the Fallopian tubes, uterus, cervix, and the cranial portion of the vagina, and the Wolffian ducts regress. The gonadotropin levels peak at midgestation and decrease toward birth and are suppressed at term. 12

The development of the primordial follicle in the fetal ovaries begins before the 13th week of gestation. The pool of primordial follicles is approximately 680,000 at the 34th week of gestation. Subsequently, the pool remains stable. 13 This pool relies on estrogen levels in the fetal circulation, which are mainly produced by the placenta. The roles of FSH and LH in ovarian development during pregnancy are not completely understood. It seems that hypothalamic stimulation is necessary to ensure physiological ovarian development after the 7th month of gestation. 14

Additionally, female fetuses produce higher LH and FSH levels than male fetuses. 15 16 Another marked difference between the sexes is that the LH levels overcome the FSH levels in male fetuses, 17 whereas the opposite situation occurs in females.

At delivery, in healthy infants, the LH and FSH levels are low in the cord blood in both sexes. 18

This complex genetic signaling pathway is not the unique determining factor of the development of sexual identity. Environmental factors also play an essential role in gonadal differentiation, and many studies have shown a link between genital malformation in humans and exposure to endocrine-disrupting chemical compounds, 19 generally related to parental occupation during pregnancy. 20 Five-year-old Vietnamese children demonstrated disruptions in androgen, including decreases in T and DHEA levels and an increase in A-dione levels, which are associated with highly chlorinated dioxin congeners. These disorders are likely due to stimulation or inhibition of the activity of steroidogenic enzymes such as CYP17 lyase, 3β-HSD, and 17β-HSD by dioxin exposure. 21

In the reported case, the patient presented two genetic mutations involved in the pathogenic pathway of the DSD condition associated with the clinical features of KS, a developmental disease that associates with HH, due to GnRH) deficiency, and anosmia, related to the absence or hypoplasia of the olfactory bulbs. Given the variable degree of hyposmia in KS, the distinction between KS and normosmic idiopathic HH is currently unclear, especially as HH patients do not always undergo detailed olfactory testing. 22 This syndrome is very rare, with an estimated prevalence of 1:80,000 in males and 1:40,000 in females. 23

The GNRHR, autosomal recessive heterozygous missense mutation, concerns the GNRHR gene that encodes the receptor for type 1 GnRH. This receptor is expressed on the surface of pituitary gonadotrope cells as well as lymphocytes, breast, ovary, and prostate. Following the binding of GnRHR, the receptor associates with G-proteins that activate a phosphatidylinositol-calcium second messenger system. Activation of the receptor ultimately causes the release of gonadotropic LH and FSH. Defects in this gene are a cause of HH. 23 24

The patient presented also an autosomal dominant heterozygous missense mutation of the *PROKR2* gene, the protein encoded by this gene is an integral membrane protein and G protein-coupled receptor for prokinetics. Prokineticins are secreted cysteine-rich proteins that possess diverse biological activities including effects on neuronal survival, gastrointestinal smooth muscle contraction, 25 and circadian locomotor rhythm. 26 Prokineticins can bind to two different G protein-coupled receptors, prokineticin receptor-1 and -2 (PROKR1 and PROKR2), which have about 85% sequence identity. PROKR2 shows relatively localized distribution in the central nervous system. 25

Moreover, the mRNA expression of PROKR2 has been detected in the subventricular zone and the olfactory bulbs.

It was found that Prokr2 −/− knockout mice exhibit early hypoplasia of the olfactory bulbs and severe atrophy of the reproductive organs in both sexes, a phenotype reminiscent of the KS features. In addition, immunohistochemical analysis of these mice revealed that the neuroendocrine GnRH cells were absent from the hypothalamus. 27

To date, digenic inheritance of KS has been shown in few patients who had monoallelic missense mutations both in *PROKR2* or *PROK2* and in other KS genes (KAL1, FGFR1) or genes underlying normosmic congenital HH (GNRHR, KISS1R). 28 29

A subgroup of patients with KS can present other clinical manifestations, such as bimanual synkinesis, abnormal ocular movements, congenital ptosis, coloboma of the iris, nasal cartilage agenesis, malformation of the external ear, cleft lip or palate, unilateral or bilateral renal agenesis, agenesis of the corpus callosum, hypodontia, skeletal anomalies of the feet and hands, and obesity. 22 30 31 Additional anomalies have so far not been reported in KS patients carrying mutations in *PROKR2* or *PROK2* , with the notable exception of a severe sleep disorder and marked obesity in one patient, 30 which could be related to the known function of prokineticin-2 signaling in behavioral circadian rhythms, including sleep–wake and ingestive behavior. 32 In the reported case, none of these associated signs and symptoms were present.

This is the only case report concerning a patient with 46 XX true hermaphroditism affected by HH and digenic inheritance of KS.

In the diagnosis process of a patient with ambiguous genital malformation, primary importance is given to an accurate anamnesis, looking for inherited forms of genital anomalies and maternal exposure to chemical compounds.

Ultrasound as well as other complementary imaging such as magnetic resonance imaging (MRI) and computed tomography as well as exploration laparoscopy helps to rule out associated anomalies and to better understand the internal genitalia development. Nevertheless, the usefulness of forebrain MRI in diagnosing the disease in children too young to undergo meaningful testing of olfaction or the hypothalamic–pituitary–gonadal axis should be emphasized, 33 even though normal olfactory bulb images have been reported in a few KS patients. 34

Endocrinology assessment is mandatory to rule out any deficiency in hormonal pathways.

Biopsies and histological studies of the surgical samples can help determine the sex of the patient; in this case, two biopsies of the left inguinoscrotal mass were carried out preoperatively, showing ovarian tissue, and the surgical specimen showed well-differentiated testis.

Moreover, genetic study and karyotype of patients with genital ambiguity are mandatory to formulate the correct diagnosis and offer appropriate therapy.

## Conclusion

In the case of sexual differentiation disorders, definite gender assignment can be challenging, and it is based on phenotype, genotype, gonadal histology, rearing gender, and social factors.

Genotypes play an important role in the decision-making process and help in understanding the individual basis of the pathogenetic origin of the condition. Nevertheless, some pathogenic pathways are not yet completely understood, as the role of gonadotropin during the embryologic development of the genital tract, and further research in families carrying these mutations is necessary to allow a better understanding of the development of sexual characters.

A multidisciplinary discussion between endocrinology, pediatrician, geneticist pediatric surgeon, and urologist is necessary to assure the patient of the best possible treatment option and gender assignment.
